# Autoimmune Neuromuscular Disorders: Emerging Insights and Future Frontiers

**DOI:** 10.3390/brainsci14030270

**Published:** 2024-03-12

**Authors:** Vincenzo Di Stefano, Filippo Brighina

**Affiliations:** Department of Biomedicine, Neuroscience, and Advanced Diagnostic (BIND), University of Palermo, 90129 Palermo, Italy; filippo.brighina@unipa.it

In recent years, our knowledge rapidly increased with respect to the immunology and immunological aspects of neuromuscular disorders [1,2]. Indeed, available antibody tests for many autoimmune conditions have driven growing interest in autoimmunity, as well as the possibility of effectively treating patients affected by the autoimmune disease using newly developed and emerging target therapies (i.e., rituximab, eculizumab, efgartigimod, etc.) for the treatment of myasthenia gravis (MG) and chronic inflammatory demyelinating polyradiculoneuropathy (CIDP) [3,4,5,6]. In particular, anti-CD20 monoclonal antibodies selectively hit B-lymphocytes, while complement inhibitors offer an effective solution in complement-mediated disorders and they represent a lifeline in refractory MG; finally, Fc-Rn inhibitors selectively reduce serum IgG levels with a wide spectrum of actions, and they are effective even in the absence of complement activation, with the possibility to treat more condition at the same time, starting a revolution for the treatment of autoimmune neurological conditions [7]. On the other side, advances in the diagnosis and evaluation of these disorders have significantly contributed to this revolution [8]. Also, the description of an immunological basis with specific diagnostic antibody testing for relevant conditions such as “Stiff person syndrome”, and other “paraneoplastic syndromes”, has directed neurologists to a new scenario in which acquired immunological neuromuscular conditions are probably overcoming degenerative neuromuscular and inherited disorders in clinical practice [9,10]. This Special Issue aims to enhance and report recent evidence on the immunological aspects of neuromuscular disorders.

## 1. Overview of Published Papers

Some papers from this Special Issue have focused on laboratory and instrumental biomarkers for diagnosis and disease progression in autoimmune neuromuscular disorders. Remarkably, inflammatory biomarkers are useful in the evaluation of inherited disease, as shown in the study conducted by Luigetti et al. In this study, increased IFN-alpha and IFN-gamma levels focused attention on the potential of cytokine profiling in hereditary transthyretin amyloidosis (Contribution 1). Regarding serum biomarkers, the value of antibody determination has been deepened in chronic dysimmune neuropathies (Contribution 2). Indeed, in many cases, the diagnosis is demanded to be in accordance with clinical and neurophysiological criteria, but there is a need for differential diagnosis in atypical cases [11].

On the other side, the description of an immunological basis with specific diagnostic antibody testing for relevant conditions, such as “Stiff person syndrome” (SPS), and other “paraneoplastic syndromes” might have contributed to the revolution in the field of neuroimmunology. Zhang et al. described the association of GAD-antibody-related focal segmental SPS and encephalitis with the demonstration of antibodies in the serum and cerebrospinal fluid in the absence of malignancy (Contribution 3). An interesting study from China described twenty-five patients with paraneoplastic syndrome associated with neuropathy showing neurophysiological patterns of peripheral nerve damage (Contribution 4). Of note, Yang et al. reported a case series of paraneoplastic amyotrophic lateral sclerosis related to anti-GAD and SOX1 antibodies to broaden the understanding of this rare and debated condition (Contribution 5).

Finally, another interesting paper investigated the frequency and clinical correlates of cognitive impairment in MG, exploring its connection with the severity of the disease or the autoimmune process (Contribution 6).

## 2. Conclusions

Autoimmune conditions represent an emergent field in neurology. Laboratory, instrumental and neurophysiological biomarkers for diagnosis, and responses to treatment are in demand. More attention should be focused on neuroimmunology in residency programs, and more research is needed to face the increasing number of drugs available to treat autoimmune neuromuscular disorders.
